# Intratumoral submicron particle docetaxel inhibits syngeneic Renca renal cancer growth and increases CD4+, CD8+, and Treg levels in peripheral blood

**DOI:** 10.1007/s10637-020-00922-5

**Published:** 2020-03-20

**Authors:** Holly A. Maulhardt, Alyson M. Marin, Gere S. diZerega

**Affiliations:** 1 US Biotest, Inc., 231 Bonetti Drive, Suite 240, San Luis Obispo, CA 93401 USA; 2 NanOlogy, LLC., 3909 Hulen Street, Fort Worth, TX 76107 USA

**Keywords:** Intratumoral, Docetaxel, NanoDoce, Renal carcinoma, Renca

## Abstract

Administration of chemotherapeutics as direct injections into tumors offers increased anti-tumor activity and reduced systemic toxicity. In this study, the Renca syngeneic murine xenograft model of renal cancer was used to evaluate the effects of intratumoral (IT) submicron particle docetaxel (NanoDoce®) on tumor growth and immunomodulation. Tumor volume (TV) was compared to controls, including intravenous (IV) chemotherapy. Flow cytometry of peripheral bloods and tumors was used to evaluate immune cell populations. Groups of animals were inoculated with a second Renca tumor at a site distant from the primary tumor. IT NanoDoce significantly reduced primary TV and reduced the growth rates of untreated secondary tumors. CD4+, CD8+ and Treg populations were increased in peripheral bloods from animals administered IT NanoDoce. Additional evaluation of the effect of IT NanoDoce on peripheral and local immune cell populations as well as the impact on sites of distant tumor growth are warranted.

## Introduction

Previously, we found that submicron particles of docetaxel (NanoDoce®) injected into UM-UC-3 and 786-O xenograft tumors in rodents significantly inhibited or eradicated the tumor as well as stimulated an immune cell infiltrate into the tumor. In the same models, similar reductions in tumor volume and immune cell infiltration into the tumor did not occur following IV docetaxel treatment. Evaluation of tumor tissues from these animals determined that IT NanoDoce administration resulted in high levels (> 1500 μg/g of tissue) of docetaxel within the tumor up to 50 days after treatment [1]. Further, we found that nebulized submicron particle paclitaxel (NanoPac®) substantially reduced or eradicated Calu-3 non-small cell lung cancer (NSCLC) tumors in immunocompromised mice which did not occur following IV administration of nab-paclitaxel [2, 3]. These previous studies were performed in rodents with genetically depleted T cells, whereas here we evaluated the response of an intact immune system to direct injection of NanoDoce compared to IV docetaxel in a Renca syngeneic model.

This report describes preliminary evaluation of efficacy, toxicity and immunomodulation in a syngeneic mouse model when NanoDoce is administered directly to a primary renal cell carcinoma. The effects on an untreated secondary tumor implanted at a site distant from the treated primary tumor are explored. Tumor growth inhibition following IT injection of NanoDoce is compared to IT vehicle control and IV chemotherapy (docetaxel) treatments. Flow cytometry is used to monitor treatment-related changes in immune cell populations in the peripheral blood and tumor microenvironment (TME). Although a large disparity in TV makes interpretation of local immunomodulation challenging, significant changes in circulating tumoricidal cells coupled with reduced distant untreated tumor growth suggests locoregional NanoDoce treatment potentiates a cytolytic secondary immune response.

## Results

### Primary and secondary tumor growth

**Fig. 1:**
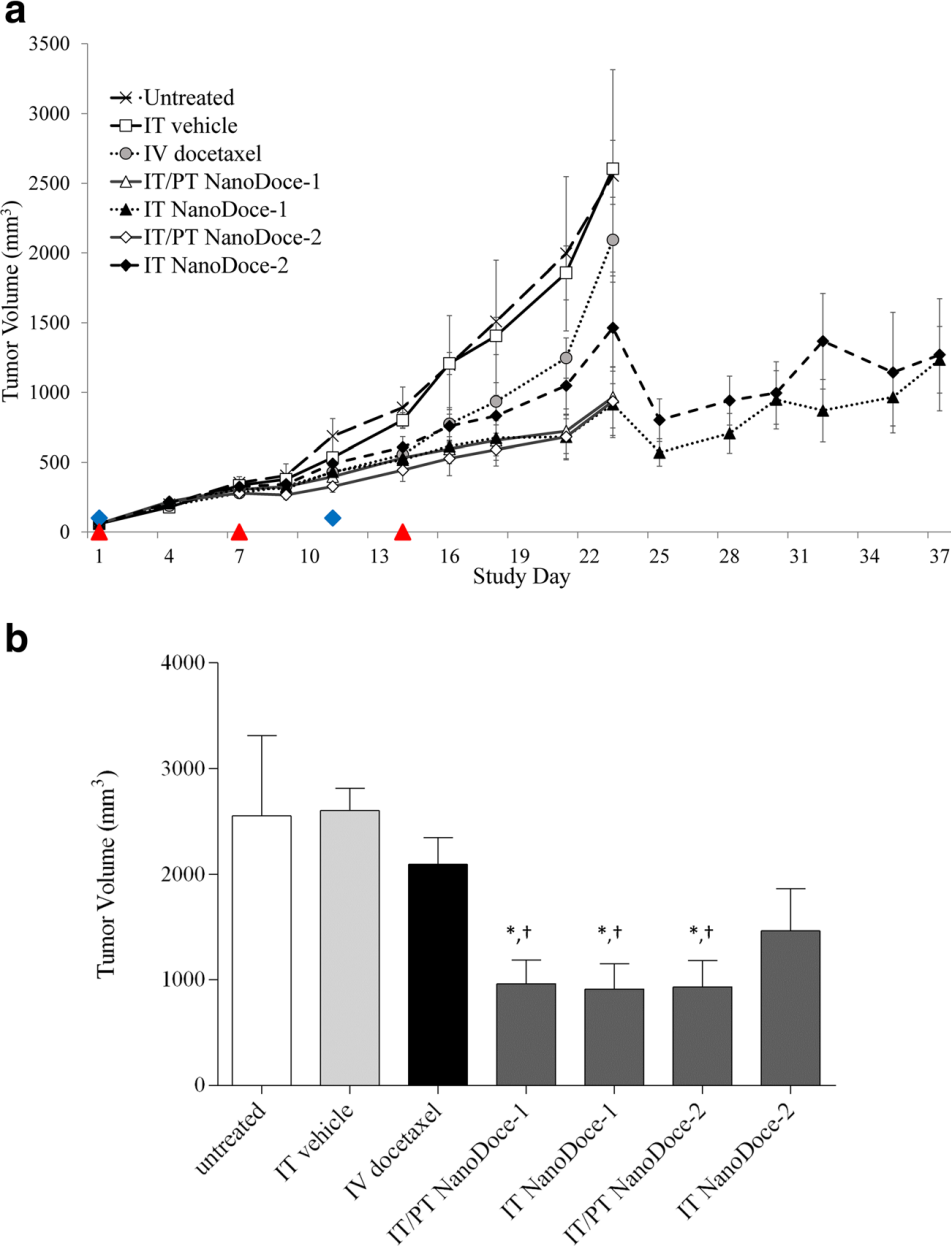
**a** MTV for animals implanted with a single tumor. All treatments were initiated on Study Day 1, 12 days after tumor implantation when MTV = 56 to 59 mm^3^. Untreated group: *n* = 6 (one tumor in the untreated group appeared to regress at Day 16 and this animal was excluded from tumor volume comparisons), all other groups: *n* = 10. IV docetaxel was administered at 10 mg/kg and 5 mg/kg on Day 1 and 11, respectively (blue diamonds). NanoDoce-1 (27.5 mg/kg) and NanoDoce-2 (55 mg/kg) treatments were locally administered on Days 1, 7, and 14 (red triangles). **b** Day 23 MTV for animals implanted with a single tumor. * = *p* < 0.001 vs. IT vehicle; † = *p* < 0.01 vs. docetaxel. Error bars = ± 1 SEM

In order to evaluate the anti-tumor activity of NanoDoce on the growth of renal cell carcinoma tumors, we treated tumor-bearing mice with IT vehicle (control group), IV docetaxel, or NanoDoce at two different doses: NanoDoce-1 (27.5 mg/kg) and NanoDoce-2 (55 mg/kg) administered IT or intratumoral/peritumoral (IT/PT) (Table 1). Tumor growth data for animals implanted with a single tumor are presented in Fig. 1a.

**Table 1:** Treatment groups

n	Number of tumors implanted	Treatment group	Dose (mg/kg)	Route of administration	Treatment days
6	1	untreated	–	–	–
10	1	vehicle^a^	–	IT^b^	1, 8, 15
10	1	docetaxel	10 and 5	IV	1, 11
10	1	NanoDoce-1	27.5	IT/PT^c^	1, 8, 15
10	1	NanoDoce-1	27.5	IT	1, 8, 15
10	1	NanoDoce-2	55	IT/PT	1, 8, 15
10	1	NanoDoce-2	55	IT	1, 8, 15
15	2	vehicle-2°	–	IT	1, 8, 15
15	2	docetaxel-2°	10 and 5	IV	1,11
15	2	NanoDoce-1-2°	27.5	IT/PT	1, 8, 15

^a^Vehicle formulation = 0.22% polysorbate 80/1.76% ethanol in saline; ^b^Intratumoral (IT) administration; ^c^Intratumoral/peritumoral (IT/PT) administration

Treated groups were followed for 23 days at which point the control vehicle group reached a mean tumor volume (MTV) of 2000 mm^3^, the defined endpoint for euthanasia. Tumors treated with locally administered NanoDoce-1 and NanoDoce-2 showed significant reduction in tumor growth compared to untreated (*n* = 5), vehicle (*n* = 10) or docetaxel (*n* = 10) treatments (Fig. 1b). Tumor growth inhibition (TGI) for animals treated with NanoDoce-1 was noticeably greater starting on Day 14 for IT/PT (*n* = 10) administration and Day 18 for IT (*n* = 10) administration. On Day 23, NanoDoce-1 administered IT/PT or IT showed median TV of 795 and 799 mm^3^ compared to 2667 mm^3^ in the vehicle control group. This represented a significant 70% TGI compared to vehicle (Fig. 1b; *p* < 0.001) and IV docetaxel (Fig. 1b; *p* < 0.01). The Day 23 MTV for NanoDoce-2 administered IT/PT (*n* = 10) was 769 mm^3^. This represented significant activity with 71% TGI compared to vehicle control (Fig. 1b; *p* < 0.001) and docetaxel (Fig. 1b; *p* < 0.01). The Day 23 MTV for IT NanoDoce-2 (*n* = 9) was not significantly reduced compared to controls.

**Fig. 2:**
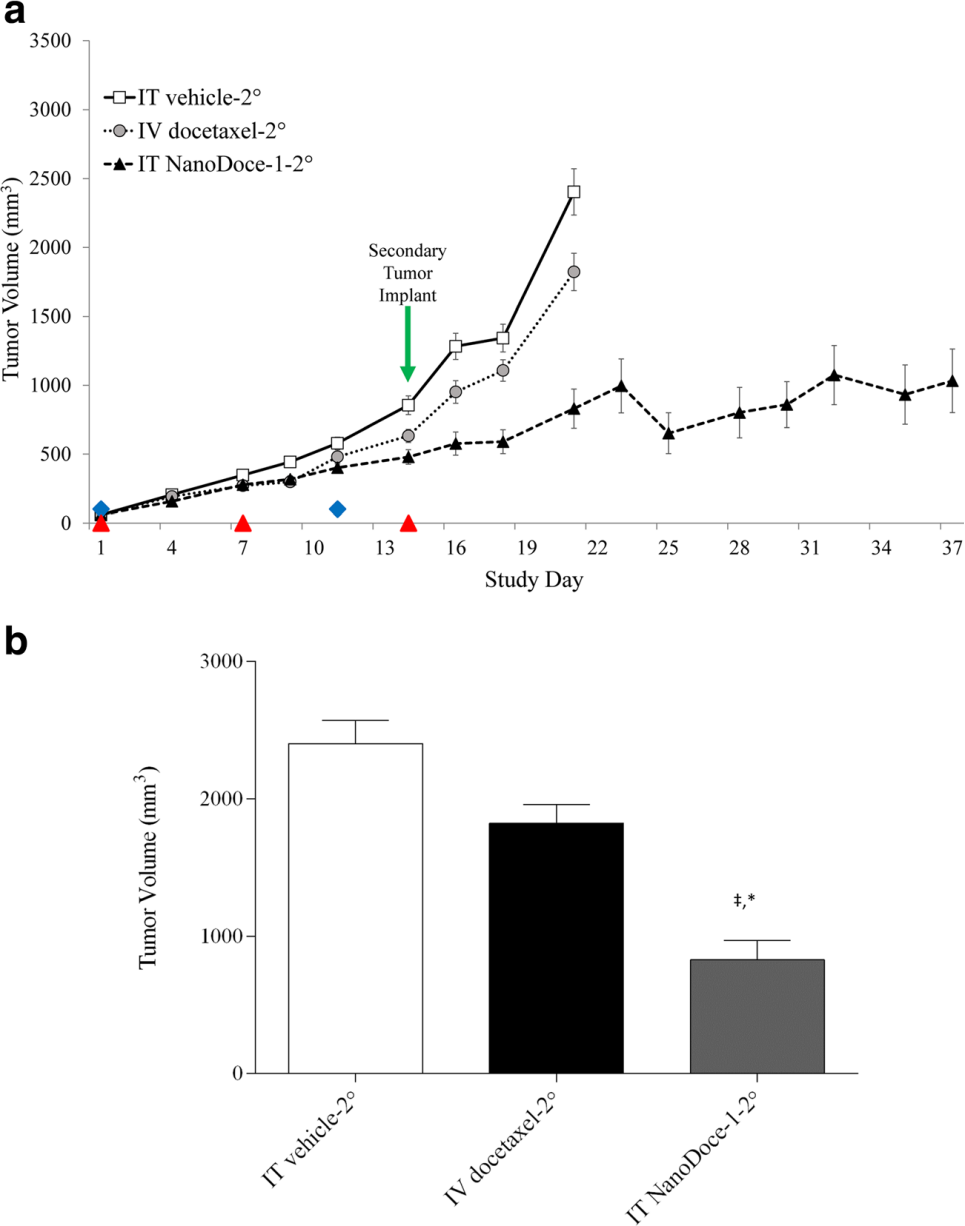
**a** MPTV for animals implanted with primary and secondary Renca tumors. All treatments were initiated on Study Day 1, 12 days after tumor implantation when group MPTVs = 58 mm^3^. IV docetaxel was administered at 10 mg/kg and 5 mg/kg on Day 1 and 11, respectively (blue diamonds). IT NanoDoce-1 (27.5 mg/kg) and IT vehicle treatments were administered on Days 1, 7, and 14 (red triangles). **b** Day 21 MPTV. ‡ = *p* < 0.0001 vs. IT vehicle; * = *p* < 0.001 vs. docetaxel. Error bars = ± 1 SEM

Three additional groups (*n* = 15/group) were established (IT vehicle-2°, IV docetaxel-2°, IT NanoDoce-1-2°) with the goal of evaluating the abscopal effect of IT therapy (Table 1). In these animals, treatment of the primary Renca tumor was initiated on Day 12 post-primary tumor implant and implant of a secondary tumor occurred 15 days later, coinciding with the final treatment of the primary tumor (Fig. 2a). Tumor growth was followed until the combined TV reached 2000 mm^3^. Due to the rapid growth of Renca tumors, the groups treated with vehicle or docetaxel reached the maximum TV between Days 21 and 23 of the study hindering the direct comparison of secondary tumor growth. Animals treated with NanoDoce-1 showed reduced secondary TV and were followed until Day 37, the last day of the study. Consistent with the results from the group bearing a single primary tumor, on Day 21 the NanoDoce-1-2° group had significantly reduced mean primary tumor volume (MPTV) compared to vehicle-2° (Fig. 2b; *p* < 0.0001) and docetaxel-2° (Fig. 2b; *p* < 0.001). These findings demonstrate that local NanoDoce treatment can effectively reduce tumor growth showing superior activity than IV docetaxel therapy.

**Fig. 3:**
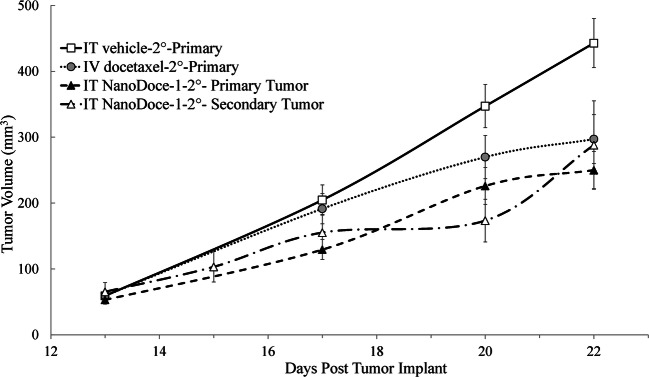
MPTV and MSTV are plotted for days 13–22 after implant, equivalent to Study Days 1–9 for primary tumors and Study Days 28–37 for secondary tumors. All primary tumor treatments were initiated on Study Day 1, 12 days after tumor implantation when MPTV = 58 mm^3^. docetaxel-2° (*n* = 15) was administered at 10 mg/kg and 5 mg/kg on Day 1 and 11, IT vehicle-2° (n = 15) and IT NanoDoce-1-2° (27.5 mg/kg; *n* = 8) treatments were administered on Days 1, 7, and 14. Secondary tumors were left untreated. Error bars = ± 1 SEM

In the IT NanoDoce-1-2° group, 8 of 15 animals had measurable secondary tumors by Day 28, 13 days after implant, and mean secondary tumor volume (MSTV) was followed for 7 additional days until animals were sacrificed (Fig. 3). Although vehicle-2° and docetaxel-2° MSTV were not available for evaluation, comparing the untreated secondary tumors to treated primary tumors from vehicle-2°, docetaxel-2°, and NanoDoce-1-2° groups suggests that untreated secondary tumors have slower initial growth rates compared to controls and similar growth rates to primary tumors treated with IT NanoDoce-1.

### Toxicity

In addition to evaluating the anti-tumor efficacy of NanoDoce, we also monitored the overall health of the animals during the study. All groups displayed mean body weight (BW) losses between 4.0 and 14.3% occurring between days 8 and 21 post-treatment initiation. Both groups treated with IV docetaxel and groups with a single tumor implant treated with IT/PT NanoDoce-1 and IT NanoDoce-2 had mean BW losses >10%. In animals administered IV docetaxel, BW losses and adverse clinical signs were severe enough to trigger dose modification such that the second cycle was administered on Day 11 (vs. as planned on Day 8), total dose was reduced from 10 to 5 mg/kg, and a planned third cycle was not administered. No dose modification was required for the NanoDoce groups.

By Day 8, tumor ulcerations characteristic of the Renca syngeneic tumor model were recorded in all groups except docetaxel-2°. No differences in rates of ulceration were noted between groups administered NanoDoce IT or IT/PT.

Prior to the point where group MTV = 2000 mm^3^, two treatment-related (TR) deaths occurred: one animal in the docetaxel-2° group was found dead on Day 6 and one animal on Day 18 in the IT/PT NanoDoce-2 group was euthanized on Day 18 due to severe BW loss. In IT NanoDoce-treated animals maintained on study beyond when control group MTV > 2000 mm^3^, one IT NanoDoce-2 was euthanized on Day 28 due to BW loss. Several animals across various treatment groups had similar necropsy findings of enlarged spleen and pale liver, kidneys and lungs at study endpoint (TV > 2000 mm^3^).

This data suggests that administering NanoDoce IT does not have overt systemic effects and that a full cycle treatment can be delivered without impacting the health of the animals. In contrast, IV docetaxel results in systemic side effects observed in the clinic, such as body weight loss.

### Treatment related immune changes

To investigate whether the increased efficacy observed in groups treated with NanoDoce was in part due to the engagement of the immune system, we analyzed blood and tumor samples obtained at endpoint (Day 23) via flow cytometry. We sampled the following groups implanted with a single Renca tumor: untreated (*n* = 6; TV range = 1437–5566 mm^3^; one animal with tumor regression excluded), IT vehicle (*n* = 10; TV range from 1437 to 3402 mm^3^), IV docetaxel (n = 10; TV range from 847 to 3072 mm^3^), IT/PT NanoDoce-1 (*n* = 10; TV range from 384 to 2746 mm^3^), IT NanoDoce-1 (*n* = 2; TV = 1568 and 2601 mm^3^), IT/PT NanoDoce-2 (*n* = 10; TV range from 172 to 2688 mm^3^) and IT NanoDoce-2 (*n* = 2; TV = 3035 and 3971 mm^3^).

**Fig. 4:**
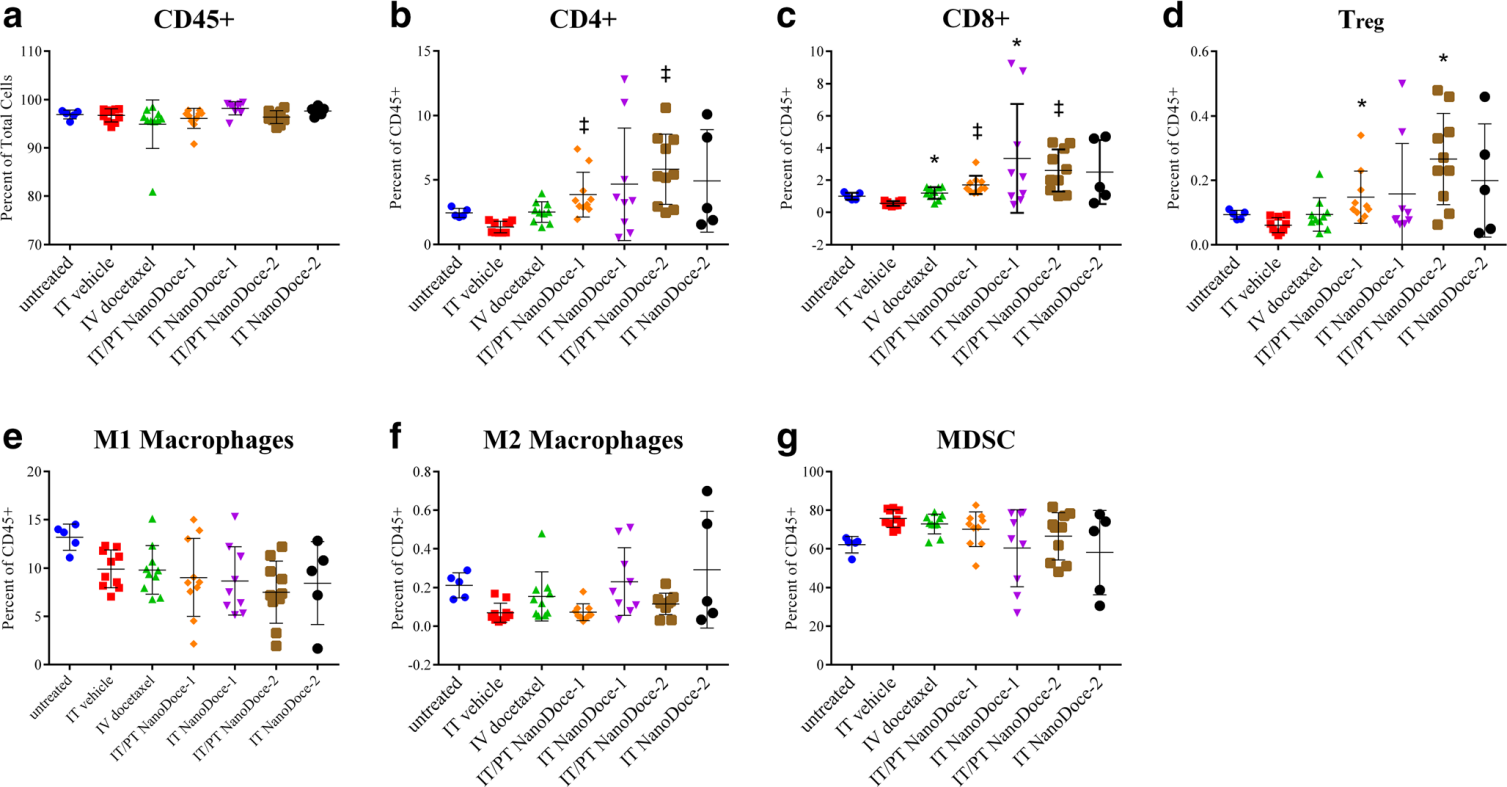
Circulating lymphocytes detected in peripheral blood 23 days after treatment initiation in animals with a single Renca tumor. **a** Similar levels of CD45+ leucocytes were detected in all samples. **b** NanoDoce treatments administered IT/PT significantly increased circulating CD4+ T cells. **c** IV docetaxel and NanoDoce treatments significantly increased circulating CD8+ T cells. **d** IT/PT NanoDoce treatments resulted in increased in circulating Treg cells. Although there was trend toward reduction following IT NanoDoce treatment, no significant differences in circulating populations of macrophages (M1 (**e**) and M2 (**f**)) or MDSCs (**g**) were detected in docetaxel or NanoDoce treatments compared to IT vehicle. Graphs show individual animal samples with center lines = means and error bars = ± Std. dev. Statistically significant differences vs. IT vehicle control are as follows: ‡ = *p* < 0.0001; * = *p* < 0.001

Peripheral blood samples from animals in all groups had similar levels of CD45+ leukocytes (Fig. 4a), whereas animals treated with NanoDoce show significantly higher circulating populations of CD4+ (Fig. 4b, *p* < 0.0001), CD8+ (Fig. 4c, *p* < 0.0001), and Treg cells (Fig. 4d, *p* < 0.001) compared to IT Vehicle. No significant differences in circulating macrophages (M1 and M2; Fig. 4e–f) or myeloid-derived suppressor cells (MDSC) (Fig. 4g) populations were detected, although a trend toward reduction of these populations was observed in animals treated with IT NanoDoce.

Tumor tissue evaluated from animals with a single Renca implant showed nonsignificant differences in percent of CD45+ cells or subpopulations. The large size and heterogeneity of TV at time of tissue collection likely contributed to the variability of flow cytometry data [4].

## Discussion

Submicron particle docetaxel (NanoDoce; CritiTech, Inc., Lawrence, KS, USA) was developed to increase IT drug residence time through local delivery. NanoDoce is produced using precipitation with a proprietary compressed antisolvent technology in which dissolved docetaxel drug substance in solution is sonicated into uniform droplets. The solvent is stripped from the droplets using supercritical fluid carbon dioxide which precipitates the pure particles of docetaxel resulting in uniform submicron particles approximately 900 nm in size. NanoDoce particles are suspended at time of use in a saline-based solution that maintains particle size and can be delivered directly to the disease site using commonly employed techniques including image-guided fine-needle injection and intravesical or intraperitoneal instillation. NanoDoce is currently being studied in a clinical trial in the local treatment of high-risk non-muscle invasive and muscle-invasive bladder cancer (NCT03636256) and development for direct injection into renal cell carcinomas (NCT04260360) is ongoing. Similarly, NanoPac (CritiTech, Inc.) is under clinical evaluation for prostate cancer (NCT04221828), ovarian cancer (NCT03029585), pancreatic cancer (NCT03077685), and pancreatic cysts (NCT03188991). Development of IT NanoPac for treatment of lung neoplasms and inhaled NanoPac for treatment of NSCLC is ongoing.

Local treatment of solid tumors with submicron particles of docetaxel or paclitaxel has the potential to overcome the limitations of conventional IV chemotherapy which include systemic exposure and rapid clearance resulting in short tumor-dwell time as well as significant systemic toxicities. IT chemotherapy provides sustained local drug concentrations over multiple cell-division cycles and results in minimal systemic toxicity, thus providing tumoricidal benefits without compromising the patient’s well-being. This was recently shown in humans by local injection of NanoPac in prostate tumors which resulted in preliminary evidence of activity without systemic toxicity in patients scheduled for radical prostatectomy (NCT03077659).

Tumor response to local injection of NanoDoce and NanoPac is significantly different compared to the IV route of administration. Direct injection of NanoDoce appears to catalyze a cascade of events involving both anti-mitotic and immune-mediated effects leading to enhanced tumor kill. In previously performed xenograft studies in immunocompromised animals, as well as here, in an immune intact syngeneic mouse model, significantly greater tumor kill was achieved following IT NanoDoce treatment compared to IV docetaxel. In the study reported here, IT NanoDoce administration also resulted in significantly increased levels of immune cells in the blood compared to controls when evaluated up to 23 days after treatment initiation.

It is well known that the TME and response of various syngeneic models to IV therapy encompasses a wide range of immunogenicity, tumor heterogeneity, and variability often relating to tumor age and size. Additionally, treatment results often differ depending on orthotopic or xenograft site of tumor cell inoculation [4, 5]. In Renca tumors, it has been found that there is a general decrease in immune cell abundance as tumors increase in size beyond 100 mm^3^ [4]. The size of the Renca tumors in the study reported here ranged from 200 to 5000 mm^3^ at the time of necropsy; leading to uninterpretable cell population data within the TME.

The inhibition of the human renal cell adenocarcinoma line 786-O [1] and the Renca murine renal cancer model by direct injection of NanoDoce versus the transient or minimal tumor reduction following IV docetaxel therapy has encouraged us to conduct a clinical trial in patients with renal cell carcinomas in which NanoDoce will be directly injected into localized tumors under image guidance. It is hypothesized that following IT NanoDoce treatments, patients with activated immune responses in the TME may develop efficacious acute and prolonged responses to immunotherapies (IO) [5]. The long residence time of NanoDoce particles may facilitate a continuous availability of tumor specific antigens allowing for prolonged immune response to initial antigens as well as reflective immune cell changes as antigens are modified. In another study evaluating a secondary Renca tumor implant, IO therapy combined synergistically with ionizing radiation resulted in nearly complete regression of the primary tumor and elicited a 66% reduction in the size of the non-treated secondary tumor [6].

The significant elevations in CD4+, CD8+ and Treg populations in peripheral blood following NanoDoce treatments suggests that circulating immune effector cells contributed to the growth inhibition of the primary tumors treated with NanoDoce and may have had an effect on the secondary untreated tumor. Although additional well-controlled studies with primary and secondary tumor implants are required, the reduced rate of secondary tumor growth coupled with the observations of increased circulating tumoricidal immune cells suggests a mechanism in which metastatic disease is controlled via response to sustained antigen exposure from persistent NanoDoce-mediated primary tumor destruction.

## Materials and methods

### Animals

Female BALB/c mice (BALB/cAnNCrl, Charles River, Morrisville, NC, USA) were 9 weeks old on study Day 1 with BW range of 16.4 to 23.6 g. Animals were fed ad libitum water and NIH 31 Modified and Irradiated Lab Diet® and were housed on irradiated Enrich-o’cobs™ bedding in static microisolators on a 12-h light cycle at 20–22 °C/40–60% humidity. On Day 8, following first tumor ulcerations, mice were individually caged to prevent trans-cannibalization of tumors.

### Tumor implantation and treatment

Renca murine renal carcinoma cells were obtained from the American Type Cell Collection (ATCC Cat. No. CRL-2947, Lot No. 58045446) and maintained as exponentially growing cultures. Cells were harvested during log phase growth and resuspended in phosphate buffered saline (PBS). Tumors were initiated by subcutaneously (SC) implanting 5 × 10^5^ cells into the right flank of each animal. Twelve days after the initial tumor implantation, designated as Day 1 of the study, animals were sorted into 10 groups such that group MTV = 58 mm^3^. On Day 15 after treatment was initiated (27 days after the first implant), three groups received a second SC implant of Renca cells into the left flank. Primary and secondary tumors were measured with calipers in two dimensions three times a week.

NanoDoce was provided by CritiTech, Inc. (Lawrence, KS. USA) as dry powder which was reconstituted at two different suspension concentrations: NanoDoce-1 = 11.0 mg/mL NanoDoce in 0.11% Polysorbate 80: 0.88% ethanol in saline; and NanoDoce-2 = 22.0 mg/mL NanoDoce in 0.22% Polysorbate 80: 1.76% ethanol in saline. Vehicle formulation was equivalent to the NanoDoce-2 formulation. Docetaxel (Taxotere®; Sigma, St. Louis, MO, USA) was used as a 20 mg/mL stock solution in 50% ethanol: 50% Tween 80.

NanoDoce-1 and NanoDoce-2 were administered IT or IT/PT in a fixed dose of 50 μL, which delivered 0.55 mg/animal and 1.1 mg/animal, respectively. Vehicle, NanoDoce-1, and NanoDoce-2 were administered IT or IT/PT; with volume split evenly across four injection sites. As Renca is known to be a highly ulcerative tumor model, IT and IT/PT administrations were included in this investigation to evaluate if the different distributions of NanoDoce resulted in differences in local toxicity. In a 20 g mouse, nominal NanoDoce doses delivered were 27.5 mg/kg (NanoDoce-1) and 55 mg/kg (NanoDoce-2). Docetaxel stock was diluted to yield 0.5 and 1 mg/mL dosing solutions in 7.5% ethanol: 7.5% polysorbate 80 in saline and was administered IV via the tail vein, adjusted to individual BW, resulting in final doses of 5 and 10 mg/kg. Primary tumors were treated according to Table 1 and secondary tumors were left untreated.

### Tumor assessment

TV was calculated using the formula in Eq. (1):

1 $$ \mathrm{TV}\ \left({\mathrm{mm}}^3\right)=\frac{\left({w}^2\ast l\right)}{2}. $$

Where *w* = width and *l* = length, in mm, of a tumor. Tumor weight was estimated with the assumption that 1 mg is equivalent to 1 mm^3^ of TV.

Percent TGI was defined as the difference between the MTV of the control group and MTV of the drug-treated group, expressed as a percentage of the MTV of the control group.

### Toxicity

Animals were weighed daily for the first 5 days and then three times a week for the duration of the study and were observed frequently for health and overt signs of any adverse TR side effects. Any individual animal with weight loss >30% for one measurement or > 25% for three measurements was euthanized and considered a TR death. A death was also considered TR if it was attributable to treatment side effects as evidenced by clinical signs and/or necropsy, or if a death was due to unknown causes during the dosing or within 14 days of the last dose. A death was classified as non-treatment related (NTR) if there was evidence that it was related to the tumor model vs. treatment.

Acceptable toxicity was defined as a group mean BW loss <20% with not more than 10% of animals in the group with TR death. Any dosing regimen resulting in greater toxicity was considered above the maximum tolerated dose and dosing was stopped. If the group mean BW loss recovered to <10% of the original, then dosing was resumed at a lower dose or a less frequent schedule.

### Flow cytometry

Twenty-three days after treatment initiation, full blood volume was collected by terminal cardiac puncture under isoflurane anesthesia, processed for whole blood with K_2_EDTA and held briefly at 4 °C prior to flow cytometry analysis. Right flank tumors were divided into two parts: one part was processed to a single cell suspension (SCS) for flow cytometry and one was formalin fixed for 24 h and transferred to 70% ethanol for preservation. Blood samples were prepared for flow cytometry by lysing red blood cells with ammonium-chloride-potassium buffer. Tumor samples were dissociated to SCS using the gentleMACS™ “Tumor Dissociation Kit” (Miltenyi Biotec; Auburn, CA, USA). Samples were filtered through a 70 μm strainer and rinsed with PBS, 2.5% fetal bovine serum (FBS). All samples (blood and tumor) were resuspended to 2 × 10^7^ cells/mL in PBS and analyzed for CD45+ lymphocytes and CD4+, CD8+, Treg, MDSC, and M1 and M2 macrophage subpopulations.

Antibodies (sourced from BioLegend (San Diego, CA, USA) and BD Biosciences (San Jose, CA, USA)) used were: anti-CD45 (clone 30-F11, # 103128), anti-CD3 (clone 17A2, #100206), anti-CD4 (clone GK1.5, #563790), anti-CD8 (clone 53–6.7, #560182), anti-CD11b (clone M1/70, #101228), anti-CD25 (clone PC61, #102036), anti-Ly-6G/Ly-6C (clone RB6-8C5, #108406), anti-FoxP3 (clone BM8, #123146), anti-CD206 (MMR, clone C068C2, #141729). 100 μL of SCS were added into 96-well plates and stained with 100 μL of Live/Dead Aqua (ThermoFisher; Waltham, MA, USA). After washing with Staining Buffer (SB), Fc receptors were blocked using TruStain FcX (BioLegend). SB containing antibodies was added to a final concentration per antibody of 0.1 μg/100 μL. Samples were stained for 30 min. at 4 °C and cells were washed and resuspended in SB. For staining of internal markers, cells were permeabilized with Transcription Factor Fixation/Permeabilization Buffer (Fisher Scientific; Pittsburgh, PA, USA). Following washes with PBS, internal marker staining was carried out using 0.1 μg of each antibody diluted in PBS. Cells were washed with PBS and resuspended in SB containing CountBright™ beads (ThermoFisher). Isotype-control antibodies were used as negative staining controls when necessary.

Flow cytometry data were collected on a LSRFortessa™ (BD Biosciences) and analyzed with FlowJo software version 10.0.7r2 (Tree Star, Inc.; Ashland, OR, USA). Cell populations were defined according to protocol and the gating strategy was determined by initial gating on singlets (FSC-H vs. FSC-A), and then live cells based on viability staining. The expression of cell surface markers was then analyzed based on cell populations of interest.

### Statistical analyses

Microsoft® Excel® for Office 365 MOS (Microsoft Corporation, Redmond, WA, USA) and Prism 6.03 (GraphPad Software; San Diego, CA, USA) for Windows were employed for graphical presentations. Prism 6.03 was employed for statistical analyses of TV and flow cytometry data. Statistical analyses of the differences between MTV in IT vehicle or IV docetaxel vs. IT NanoDoce groups were accomplished using one-way ANOVA followed by the Mann Whitney U test and *p* values <0.01 are reported. Statistical analysis of differences in cell populations in the flow cytometry data were accomplished using one-way ANOVA (Kruskal-Wallis test) followed by the Mann Whitney U test and *p* values <0.001 are reported. The two-tailed statistical analyses were not adjusted for multiple comparisons.

## Conclusions

Docetaxel (DTX, Taxotere, Docefrez™) is a standard IV chemotherapeutic approved for use in the treatment of NSCLC, gastric, head and neck, ovarian, prostate and breast cancers [7]. Clinical trials are underway to explore the use of IV docetaxel alone or in combination with other agents against other advanced solid tumors and genitourinary cancers, including renal cell carcinoma [8]. Docetaxel stabilizes microtubule polymers, inhibiting mitosis and tumor cell proliferation and impacting inflammation and immunity [9, 10]. Significant toxicities, especially neutropenia, are frequently seen in patients treated with IV docetaxel [7]. For this reason, new formulations designed to deliver docetaxel directly to tumors and avoid systemic toxicity are the subject of intense investigation. In this study, NanoDoce, submicron particle docetaxel, administered as a direct IT injection, showed significantly improved reduction in Renca tumor growth compared to IV docetaxel. Increases in tumoricidal immune cell populations as well as a reduced growth rate in an untreated secondary Renca tumor implant, suggest that the IT NanoDoce-initiated and -sustained destruction of a primary renal tumor may lead to an immune response that has the potential to decrease distant tumor growth. Further research is warranted to evaluate IT NanoDoce alone or in combination with immuno-oncology therapy in the treatment of metastatic renal cancer.

## Open access

This article is distributed under the terms of the Creative Commons Attribution 4.0 International License (http://creativecommons.org/licenses/by/4.0/), which permits unrestricted use, distribution, and reproduction in any medium, provided you give appropriate credit to the original author(s) and the source, provide a link to the Creative Commons license, and indicate if changes were made.
